# Supplementary material for: A Humanized Yeast Phenomic Model of Deoxycytidine Kinase to Predict Genetic Buffering of Nucleoside Analog Cytotoxicity
Source: Genes (Basel). 2019 Sep 30;10(10):770. doi: 10.3390/genes10100770 (PMC6826991; doi:10.3390/genes10100770)
Supplement: Supplementary file 1 [file genes-10-00770-s001.zip › Additional_Files/Additional_File7_GOTermHeatmaps.zip!/Additional_File7_GOTermHeatmaps/regulation_of_ER_to_Golgi_vesicle-mediated_transport.pdf]

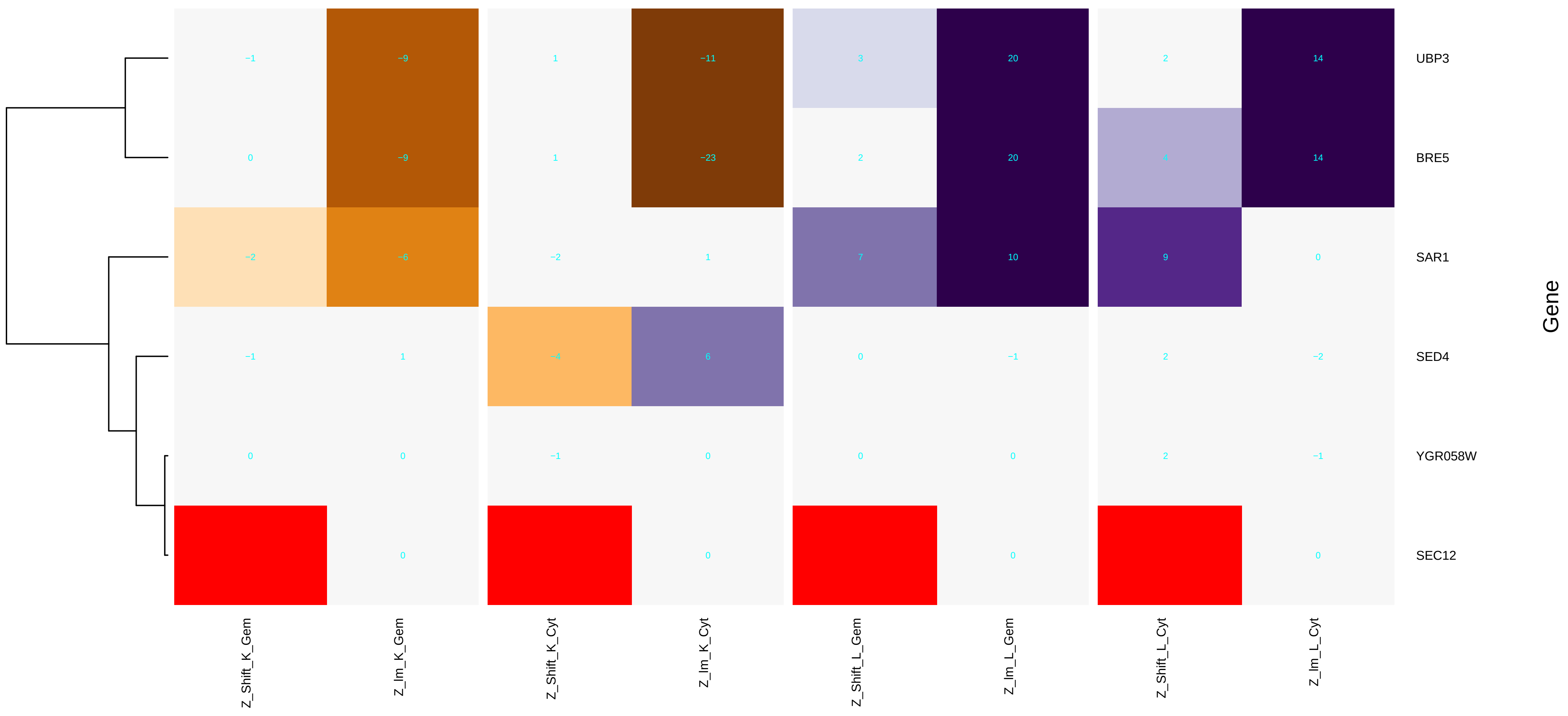
UBP3
BRE5
SAR1
SED4
YGR058W
SEC12
Gene
Z_Shift_K_Gem
Z_Im_K_Gem
Z_Shift_K_Cyt
Z_Im_K_Cyt
Z_Shift_L_Gem
Z_Im_L_Gem
Z_Shift_L_Cyt
Z_Im_L_Cyt
−1
−9
1
−11
3
20
2
14
0
−9
1
−23
2
20
4
14
−2
−6
−2
1
7
10
9
0
−1
1
−4
6
0
−1
2
−2
0
0
−1
0
0
0
2
−1
0
0
0
0
